# Light-Driven Topological Relaxation and Dynamic Scaling in Photoresponsive Polymer Films

**DOI:** 10.1021/acsphotonics.5c03137

**Published:** 2026-05-16

**Authors:** Michael de Oliveira, Sara Moujdi, Stefano Chiodini, Fabio Borbone, Antonio Ambrosio

**Affiliations:** † Center for Nano Science and Technology, Fondazione Istituto Italiano di Tecnologia, Via Rubattino 81, Milano 20134, Italy; ‡ Department of Chemical Sciences, University of Naples “Federico II”, Via Cintia, Naples 80126, Italy

**Keywords:** azopolymers, pattern formation, self-organization, topological defects, defect annihilation

## Abstract

Across nature, when systems are driven far from equilibrium, topological defects emerge as universal signatures of how order emerges from disorder. Their formation reflects the interplay between the competing time scales of driving and relaxation that govern their self-organization. Here, we investigate this process in light-responsive azopolymer films that spontaneously self-organize under continuous illumination through photoisomerization and phase separation. By tracking the spatiotemporal evolution of the emerging surface morphology, we identify a characteristic freeze-out time—the intrinsic time scale over which the initially uniform film transitions to an ordered pattern. Correlating this time scale with the defect density reveals a robust positive power-law scaling, where slower ordering near the illumination threshold yields higher defect densities, while stronger excitation accelerates domain formation and produces more coherent, defect-sparse patterns. This behavior is consistent with a self-limited regime in which pattern formation is governed primarily by the material’s intrinsic photomechanical relaxation rather than by externally imposed rates. Following the initial pattern formation, we observe a secondary self-annealing regime mediated by transient vortex–antivortex pairing that drives further defect annihilation and surface refinement. These results establish light-driven azopolymers as a versatile and fully optical platform for probing universal kinetic laws of defect evolution, dynamic topological ordering, and nonequilibrium relaxation in soft photonic materials.

## Graphical abstract

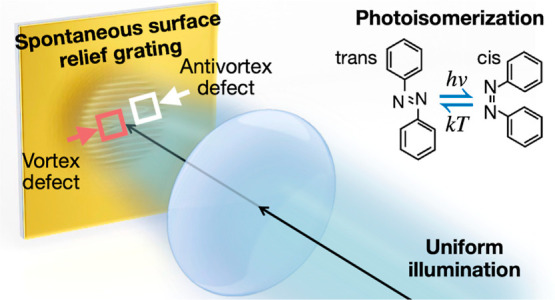

## Introduction

From the emergence of cosmic structure in the early universe^1,2^ to the symmetry breaking of superfluids,^3,4^ superconductors,^5,6^ and soft matter,^7^ nature repeatedly demonstrates how order can arise from disorder under far-from-equilibrium conditions. A unifying signature of these transitions is the spontaneous generation of topological defects—localized disruptions of order that form when a system can no longer relax adiabatically as it crosses an instability.^8^ These defects act as dynamic fingerprints of how the system negotiates the competing time scales of driving, relaxation, and noise.^9,10^

In many continuous or weakly first-order transitions, diverging relaxation times near an instability threshold can drive the system out of equilibrium, imprinting the memory of its evolution onto the final defect landscape. This principle forms the basis of the Kibble–Zurek mechanism (KZM), which connects defect formation to the breakdown of adiabaticity during symmetry breaking.^1,2^ In its canonical form, KZM predicts a power-law dependence between the defect density and the quench rate, with slower transitions through the critical point allowing more time for relaxation, resulting in fewer defects.^8^ Within the mean-field approximation for two-dimensional systems, where the average defect spacing corresponds to the correlation length, the scaling exponent takes the characteristic value of −1/2.^11,12^ The negative exponent thus encapsulates the intuitive expectation that slower quenches yield smoother, more ordered states. Although this behavior has been experimentally verified across various platforms—from cosmology^1,2^ and liquid crystals,^13,14^ to trapped ions^15,16^ and ultracold gases^17^—it also motivates a broader perspective on how order emerges when a system is driven instantaneously far from equilibrium.

In realistic driven media, external quenches are not always the dominant control parameter. Instead, the kinetics of pattern formation is often governed by the intrinsic relaxation dynamics of the material itself. As an initially uniform state becomes unstable under constant forcing, microscopic fluctuations grow, domains compete, and new spatial symmetries emerge. The characteristic time at which order first appears—the freeze-out time—and the resulting defect density together encode how the system explores its underlying energy landscape and dissipative pathways. In such regimes, counterintuitive scaling behaviors can emerge: disorder may increase rather than decrease with slower internal dynamics, as stochasticity, noise strength, and system–bath coupling reshape the path toward equilibrium. Understanding these internal dynamical links is crucial for controlling self-organization in photoresponsive, fluidic, and elastic systems, with implications for disorder management in technologies ranging from quantum annealing^18^ and neuromorphic computing^19^ to soft-matter photonics.^20^

Photoresponsive soft materials provide a particularly versatile platform for probing these far-from-equilibrium processes, where light acts simultaneously as the driving field and diagnostic probe of structural evolution. Among them, azobenzene-functionalized polymers (azopolymers) are well-known for their rich photomechanical response^21–23^ and spontaneous light-driven pattern formation.^24–26^ Under uniform illumination, azopolymer films are understood to undergo light-driven compositional reorganization, compatible with a local phase separation within the polymer matrix into immiscible *cis*-rich and *trans*-rich regions.^27^ This in turn is accompanied by internal stress and mass transport, which can destabilize the initially flat film and give rise to spontaneous surface modulations once a critical intensity is exceeded (see Figure 1a). At low illumination intensity, the photon density is small, so both the trans–cis photoisomerization and the reverse cis–trans relaxation are weak, resulting in slow, weakly correlated domain growth with broad interfaces. As intensity increases, photoisomerization becomes more efficient, promoting the formation of better-defined domains with sharper boundaries. At the same time, the connectivity between structural features decreases and the domains locally undergo a continuous-to-discontinuous transition. Under linearly polarized illumination, the domains become elongated, and the resulting surface-relief grating forms with a wave vector parallel to the polarization direction of the writing beam (see Supporting Information, Section S1), reflecting the vectorial coupling of the photoisomerization process.^22,28–30^ As the pattern develops, defects in the form of dislocations and disclinations emerge as topological markers of the nonequilibrium trajectory of the film.

**Figure 1:**
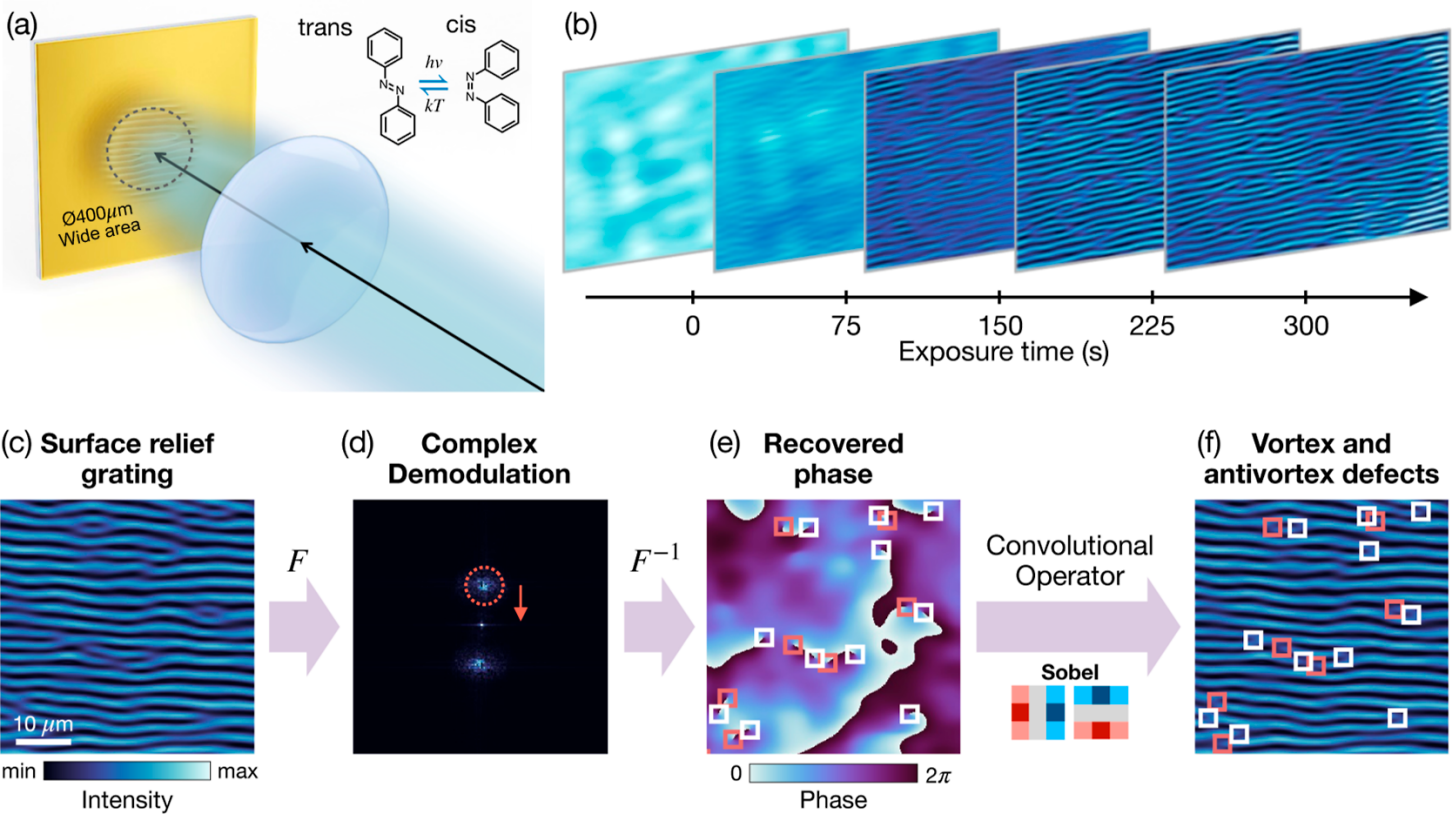
Light-driven structuring and topological defect analysis in azopolymer films. (a) A weakly focused laser beam illuminates the free surface of an azopolymer film, inducing periodic surface modulation interspersed with topological defects. (b) Time-lapse images showing the evolution of surface morphology, where periodic ripples form alongside pitchfork-like defects, some of which annihilate during continued exposure. To quantify defect dynamics, we apply complex demodulation to the (c) captured intensity images of the sample surface. (d) The corresponding Fourier transform reveals distinct side peaks. The complex envelope Ψ associated with the modulated sample surface is reconstructed by digitally isolating one of the side peaks, shifting it to the center, and performing an inverse Fourier transform. (e) Defects appear as phase dislocations in the retrieved phase map arg (Ψ), i.e., charge-1 vortices characterized by a 2π azimuthal phase winding. (f) Defect cores identified by applying a Sobel convolution operator to the phase gradient and color-coded by topological charge (vortices in red and antivortices in white).

Here, we investigate light-driven azopolymers as a model platform for studying dynamic scaling in optically driven pattern formation. While defect formation has been reported in related polymer and elastic-film settings,^31,32^ here we provide a systematic, real-time experimental characterization of defect nucleation, motion, and annihilation during surface-relief grating formation. By performing near-instantaneous optical quenches and tracking the full spatiotemporal evolution of the emerging surface morphology, we extract the freeze-out time—a characteristic delay between the onset of illumination and global pattern organization—and correlate it with the steady-state defect density. We uncover a robust positive scaling between these quantities: shallow quenches near threshold produce slow, spatially asynchronous growth with many defects, while deeper quenches drive faster, coherent ordering with fewer defects. This scaling behavior is consistent with a self-limited regime, in which defect statistics are governed predominantly by the material’s intrinsic photomechanical relaxation rather than by externally imposed sweep rates, as in KZM-type scenarios. After initial pattern formation, we observe a subsequent self-annealing regime, characterized by transient vortex–antivortex defect pairs that nucleate and annihilate during continued relaxation. Together, these results link the phenomenology of defect-mediated ordering in light-driven soft materials to the broader context of critical dynamics, highlighting azopolymers as an optically reconfigurable platform for exploring universal kinetic laws of pattern formation and nonequilibrium relaxation.

## Real-Time Defect Tracking

Experiments were performed using a linearly polarized continuous-wave 488 nm laser to induce surface patterning in azopolymer thin films. The beam was weakly focused by a 100 mm lens to illuminate a ∼400 μm-wide region on the free surface of the sample. The sample consisted of a 1 μm-thick azopolymer film spin-coated onto a 125 μm microscope coverslip; details of the polymer synthesis can be found in ref 30. To monitor the surface evolution in real time, a low-power 633 nm He–Ne probe beam was coaligned via a dichroic mirror. The surface was imaged with a 50× long-working-distance objective and a 200 mm tube lens. A long-pass optical filter blocks the writing beam, ensuring that only the probe signal is recorded. The imaging region spanned a 75 × 100 μm^2^ region at the center of the illumination spot, where the beam profile is uniform. The power of the writing beam was varied between 5 mW and 50 mW, corresponding to an approximate intensity between 4 W/cm^2^ and 40 W/cm^2^. For each exposure, the sample was laterally translated to a fresh region of the polymer film, while the illumination and imaging system remain fixed and aligned. A schematic of the setup and additional technical details are provided in the Supporting Information, Section S1. Figure 1b shows the typical time series of the evolving surface morphology under these conditions. Periodic ripple formation interspersed with pitchfork-like topological defects are clearly visible, some of which annihilate over time with continued illumination. Reference videos and atomic force microscopy images of the final sample morphology are included in the Supporting Information.

To quantitatively analyze the surface evolution and defect dynamics, we model the surface modulation as a locally periodic pattern with a carrier wavevector **q**_0_ and a slowly varying complex envelope, Ψ­(**r**) = *A*(**r**)*e* ^*i*ϕ(**r**)^, where |Ψ| = *A* encodes the local modulation amplitude and arg (Ψ) = ϕ encodes the local phase. We employ complex demodulation to retrieve Ψ­(**r**), as illustrated in Figure 1c–f. The recorded intensity images are first Fourier transformed to obtain their spatial-frequency representation (Figure 1d). Because the emerging morphology is approximated by a rectified sinusoid (see Figure S2), the Fourier spectrum is dominated by two first-order sidebands centered at ± **q**_0_, corresponding to the periodic surface modulation.

To reconstruct the slowly varying complex envelope Ψ­(**r**), we isolate one sideband (a small window around + **q**_0_), shift it to the center of the Fourier plane, and apply an inverse Fourier transform. This is a spatial analogue of single-sideband demodulation, and it recovers the complex envelope without requiring an external reference beam. The defects appear as zeros of |Ψ| accompanied by 2π phase winding in arg (Ψ) (as shown in Figure 1e), corresponding to local phase dislocations of the surface pattern^33^ (not vortices in the incident optical field). In a separate study, we show the mechanical implications of such defects in terms of local elasticity.^34^

To identify and track the defects—often numerous in a single frame—we developed a custom analysis routine based on spatial convolution.^35^ Specifically, a Sobel operator is applied to the reconstructed phase map to enhance local phase gradients and highlight phase singularities (more details can be found in Supporting Information, Section S2). Each defect is labeled by its topological charge—positive (vortex) or negative (antivortex)—and overlaid on the original image (Figure 1f). This allows us to follow individual defect events (nucleation, drift, and annihilation) over time across many realizations, rather than inferring dynamics from steady-state snapshots. These real-time measurements form the basis for the statistical analyses presented in the subsequent sections.

## Results and Discussion

To investigate how light-induced instabilities give rise to ordered surface patterns, we monitored the real-time evolution of azopolymer films under continuous spatially uniform illumination. Above a critical excitation threshold, the initially uniform azopolymer film enters a driven unstable regime in which a spatially periodic surface modulation spontaneously nucleates and grows and evolves under coupled photoisomerization and elastic relaxation. The emerging modulation is driven by the local gradient of the concentration of the two isomers, which triggers spontaneous symmetry breaking and introduces spatial variations in the local phase of the pattern. As the dominant grating mode grows, the phase cannot relax smoothly everywhere and topological defects (phase dislocations) naturally emerge in the surface grating. These defects provide a useful fingerprint of how the system negotiates competing time scales of driving and relaxation during the formation of global spatial order (i.e., increasing spatial coherence of the periodic modulation).

Operationally, we use the first-order diffraction efficiency of the developing surface grating as an experimental measure for the order parameter of the system. This choice is motivated by the fact that diffraction at the dominant carrier wavevector **q**_0_ is governed not only by the local modulation depth but also by how well the periodic modulation is phase aligned across the illuminated area. In a highly ordered grating (in the absence of defects), contributions at ± **q**_0_ add coherently across the illuminated area, and the diffracted power is maximized. Conversely, phase disorder and topological defects reduce this coherence, so different regions contribute with mismatched phases, suppressing coherent build-up and lowering the diffraction efficiency. In the Fourier spectrum, the emergent pattern produces conjugate first-order sidebands at ± **q**_0_, with approximately equal diffracted power in each sideband. Our complex-demodulation procedure isolates one of these sidebands and reconstructs the grating envelope Ψ­(**r**), such that spatially integrating the corresponding sideband intensity |Ψ­(**r**)|^2^ yields the diffraction efficiency. Diffraction efficiency therefore provides a physically meaningful experimental measure of global spatial order: it grows as the periodic modulation develops and approaches saturation as the pattern becomes spatially coherent (long-range order is established), as observed in Figure 2a.

**Figure 2:**
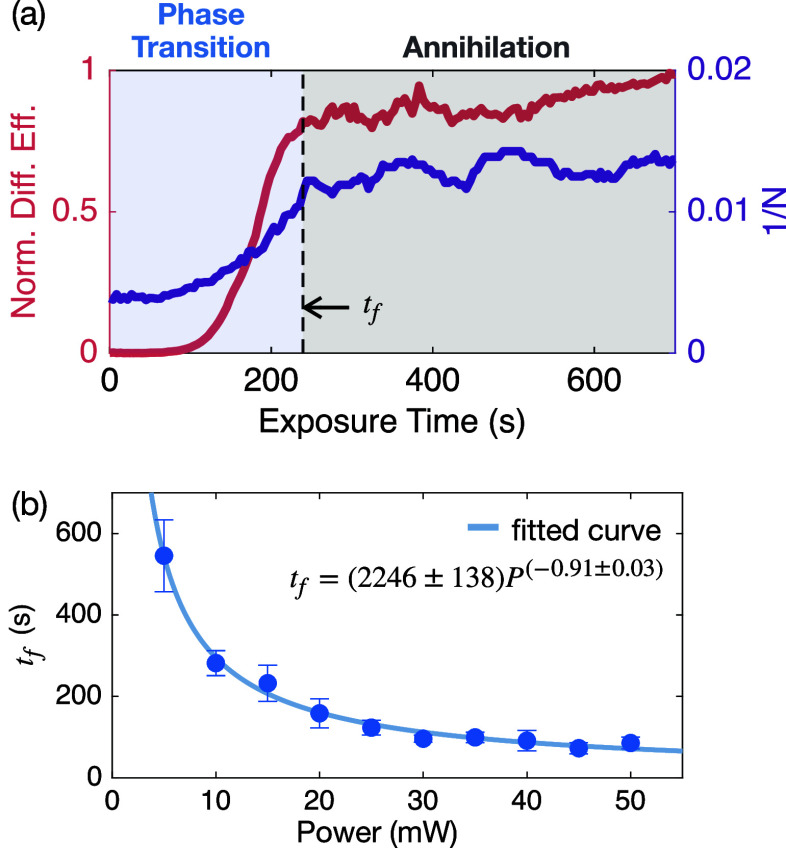
Order scaling dynamics and defect evolution. (a) Representative azopolymer dynamics for 15 mW illumination. The inverse defect count per frame (purple) and diffraction efficiency of the periodic surface pattern (red) are plotted versus exposure time. The inflection point (black dashed line) defines the freeze-out time *t*_f_, marking the onset of coarsening. Beyond *t*_f_, defect density decreases and diffraction efficiency increases linearly, indicating a secondary regime of defect annihilation and morphological refinement. (b) Dependence of *t*_f_ on illumination power, showing a monotonic decrease with increasing power. Error bars denote standard deviation over nine repeated measurements per illumination power.

We extract the freeze-out time, *t*_f_, from the inflection point of the diffraction efficiency curve (the moment when the order parameter grows most rapidly before approaching saturation), providing a reproducible, measurement-defined time scale for when the pattern is largely established (Figure 2a). Importantly, *t*_f_ is a macroscopic ordering time (tens to hundreds of seconds under our conditions) distinct from the intrinsic photoisomerization time. It therefore defines the characteristic time scale over which order emerges following the onset of the optical instability, indicating that much of the surface modulation develops within this window. Averaging over nine independent realizations for each illumination power yields a consistent measure of the transition time scale (Figure 2a; full data in Figure S4).

In parallel, we monitor the spatiotemporal evolution of the defect density, quantified by the inverse number of defects (1/*N*) per frame. The resulting defect curves exhibit a sigmoidal shape, with a clear inflection point marking the maximum rate of defect annihilation. Notably, this point coincides with *t*_f_, confirming that both order growth and defect reduction are governed by the same intrinsic time scale. This correspondence is expected because both observables derive from the same evolving grating order parameter Ψ: the diffraction efficiency depends on the magnitude |Ψ|, whereas the defect count is extracted from the phase arg (Ψ). Physically, we interpret *t*_f_ as marking the onset of global pattern organization and crossover from the initial growth stage toward coarsening-dominated dynamics. Beyond *t*_f_, the number of defects decreases gradually while the diffraction efficiency continues to rise nearly linearly, revealing a secondary slower relaxation regime dominated by defect interactions and surface refinement. We return to this regime in more detail below.

As shown in Figure 2b, the freeze-out time decreases monotonically with increasing illumination power, consistent with faster light-driven molecular rearrangement and pattern development at higher powers. Across the explored illumination powers, the extracted freeze-out time spans ∼70–500s (Figures 2b and S5), and throughout we use “faster” (“slower”) ordering to refer to smaller (larger) *t*_f_, respectively. This trend is consistent with previous observations of light-driven pattern formation in azopolymers^27^ and aligns with known photophysical saturation effects. Furthermore, the illumination intensity provides a tunable control parameter not only governing the characteristic freeze-out time and resulting defect density but also adjusting the periodicity of the final surface relief (see Supporting Information, Section S3), enabling a route to reconfigurable topographic control.

Plotting the defect number *N* against the freeze-out time *t*_f_ on a log–log scale (Figure 3) reveals a clear power-law dependence $N\propto {\left(t_{f}\right)}^{\alpha }$ with a positive exponent α = (+0.52 ± 0.05). The magnitude of this slope is comparable to that typically reported in Kibble–Zurek analyses, but with the opposite sign—indicating that longer freeze-out times correspond to higher defect densities. We emphasize that this resemblance is purely empirical: the inverted scaling arises from the material’s intrinsic photomechanical relaxation and nonlinear saturation, rather than from any violation of Kibble–Zurek causality. Direct imaging of the azopolymer surface supports this result, showing that stronger illumination power (deeper quenches with shorter *t*_f_) produces smoother morphologies with fewer defects, whereas near-threshold illumination (shallower quenches with longer *t*_f_) leads to highly fragmented textures with a dense defect network (Figure S5).

**Figure 3:**
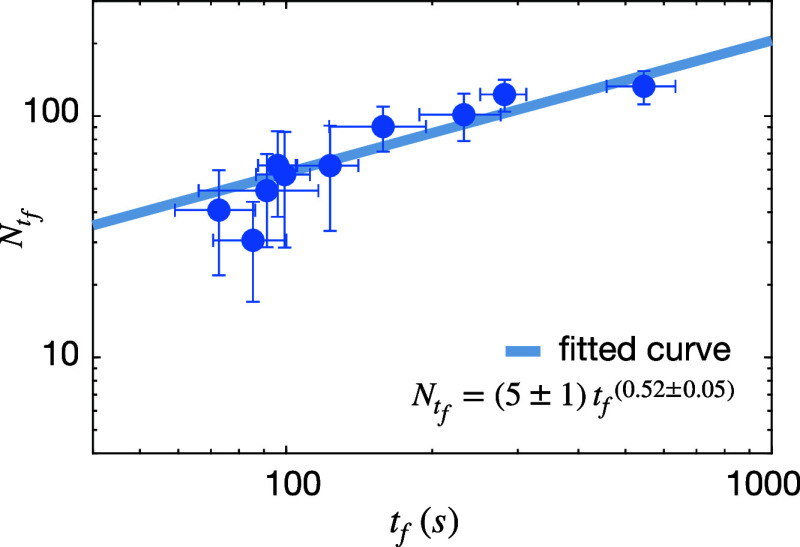
Power-law scaling between defect density and freeze-out time. Log–log plot of the steady-state defect number *N* versus freeze-out time *t*_f_. Error bars denote standard deviation over nine repeated measurements per illumination power.

The observed increase in defect density with longer freeze-out times provides insight into the fundamental kinetics of pattern formation near an instability threshold. When a uniform azopolymer film is illuminated slightly above the critical intensity (a shallow quench), the growth rate of random fluctuations is small. Different regions likely fall out of equilibrium at slightly different times, and the pattern nucleates from many uncorrelated domains, producing a fragmented texture that is rich in defects. By contrast, for deeper quenches (at higher excitation above threshold), fluctuations grow faster, and the onset is more synchronous across the film, yielding more coherent patterns with fewer defects.

This counterintuitive trend, where slower ordering leads to greater disorder, is consistent with a self-limited regime. In such regimes, common to hydrodynamic,^9^ chemical,^36^ and optical^37^ pattern-forming systems, the external drive instantaneously thrusts the medium far from equilibrium, leaving the emergence of order governed by its intrinsic relaxation dynamics rather than the external quench rate. The resulting behavior contrasts with causality-limited transitions, such as those described by the Kibble–Zurek mechanism, where slower sweeps across a critical point allow adiabatic evolution and thus yield fewer defects. Here, the optical excitation acts as an effectively instantaneous quench relative to the observed ordering dynamics, so the defect statistics primarily capture the intrinsic growth and coarsening rather than a breakdown of adiabaticity during a slow sweep.

The observed inversion suggests that the relevant freeze-out is governed less by causality-limited dynamics and more by the competition between photochemical driving, mass transport, and mechanical relaxation (i.e., by how efficiently the film can reorganize while it is being driven). Our data would be consistent with a scenario in which illumination modifies molecular mobility and cis–trans redistribution, enabling mass transport and the growth of a surface modulation. As the surface modulation develops, the evolving topography (height gradients and curvature) may introduce viscoelastic stresses in the polymer matrix, providing an additional relaxation channel that can couple spatially separated regions. If this mechanical relaxation is slow compared with the photochemically driven reorganization, such constraints could impede coarsening and defect annihilation, leading to a higher defect density. Although these stresses are not directly measured here, this interpretation is consistent with prior work on instability-driven azopolymer patterning and modeling approaches that incorporate coupled composition and mechanical effects (elastic penalties) during light-driven phase separation.^27^ More direct tests of this picture could be obtained by combining the present measurements with independent probes of mechanical response (e.g., viscoelastic or stress mapping).

After this initial pattern formation stage, the system enters a slower secondary relaxation regime characterized by defect motion and annihilation. In this regime, the surface morphology continues to evolve as defects move, interact, and annihilate, consistent with continued coarsening under photochemical driving and viscoelastic relaxation. Although the analogy to other topological defect systems is qualitative, this observation underscores how light can both generate and erase defects within a single experimental setting. As shown in Figure 4a, the defect density decreases approximately linearly with time, consistent with the pairwise annihilation of oppositely charged defects, while the global balance between positive and negative charges remains conserved. The accompanying rise in diffraction efficiency is consistent with the gradual refinement of the surface grating as defects annihilate and the spatial coherence of the surface modulation improves (Figure 2a).

**Figure 4:**
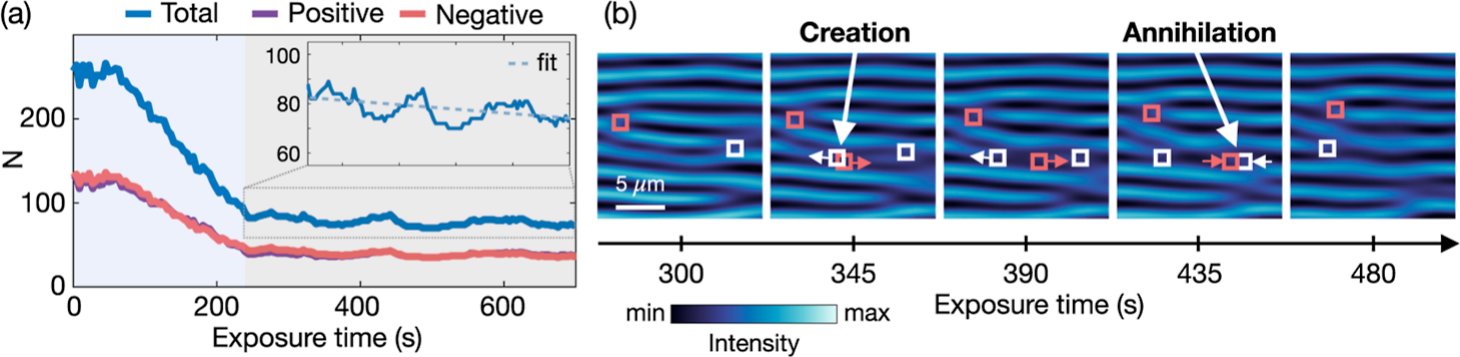
Defect dynamics and self-annealing following initial pattern formation. (a) Time evolution of vortex (purple) and antivortex (red) defect number for 15 mW illumination power, showing global charge conservation. Inset shows the total number of defects (blue) decreases approximately linearly, driven by the mutual annihilation of vortex–antivortex defect pairs. (b) Image sequence showing spontaneous nucleation of short-lived vortex–antivortex defect pairs near existing defects. These transient pairs are observed during late-stage relaxation and may assist annihilation in regions where direct recombination is hindered.

Interestingly, this relaxation process is not strictly monotonic. As illustrated in Figure 4b, we observe the spontaneous appearance of short-lived defect pairs, typically nucleating near preexisting defects. At the level of our observations, these events dominate during a late-time relaxation regime (after the primary pattern has formed), and we therefore interpret them as part of a secondary defect relaxation strategy to a more ordered steady state, rather than as a renewed global symmetry-breaking transition. One plausible interpretation is that, in regions where direct annihilation is energetically or geometrically constrained, the system may temporarily create localized disorder (new vortex–antivortex defect pairs) to facilitate the recombination of persistent defects and reduce residual morphological frustration. This behavior resembles relaxation strategies observed in other far-from-equilibrium systems, such as vortex recombination in two-dimensional superfluids and defect coarsening in active nematics and liquid crystals. Here, however, the same qualitative phenomenology arises in a light-driven soft film under continuous optical activation; developing a predictive system-specific model for these events is an important direction for future work.

## Conclusion

Our findings establish light-driven, spontaneous surface structuring in azopolymers as a versatile platform for studying nonequilibrium relaxation and defect-mediated ordering in soft photonic materials. We uncover a reproducible dynamic scaling regime in which the density of topological defects increases with the intrinsic time scale over which order emerges following optical excitation. This behavior, in contrast to causality-limited frameworks such as the Kibble–Zurek mechanism, supports the interpretation of a self-limited regime where pattern formation is governed predominantly by the internal competition between photoisomerization, mass-transport, and elastic relaxation, rather than by the rate of external driving. Beyond the initial ordering, we observe a secondary, slower relaxation pathway involving transient vortex–antivortex nucleation and annihilation. Such behavior resonates qualitatively with fluctuation-driven ordering processes observed in other systems, from cosmological structure formation^2^ and quantum criticality^38^ to active matter,^39^ highlighting the potential of our system as an accessible experimental platform for exploring broader nonequilibrium dynamics.

Beyond its conceptual value, our framework offers a practical route for the dynamic optical control of spontaneous self-organized surface morphology. By tuning the illumination intensity, one can control the freeze-out time and thereby statistically engineer the defect landscape from nearly coherent gratings to deliberately disordered photonic textures. This ability to control defect density, pattern coherence, and relaxation dynamics unlocks new opportunities for defect engineering in soft photonic materials from reconfigurable optical components to self-organizing textures. This extends to the patterning of large-area polymeric scaffolds with tailored functionalities for integrated mechanical support and graded stiffness.^34^ More broadly, our findings challenge the long-held assumption that slower ordering universally promotes fewer defects, with implications for fields ranging from quantum annealing,^20^ neuromorphic computing,^19^ to materials design.

## Supplementary Material





## Notes

Experimental setup and real-time optical monitoring; complex demodulation and extraction of topological defect positions from phase maps; AFM characterization of power-dependent surface structuring and pitch tunability; additional diffraction-efficiency and defect-dynamics data across illumination powers; and morphology snapshots at the freeze-out time (PDF)
Light-driven surface evolution (MP4)
