# Supplementary material for: Light-Driven Topological Relaxation and Dynamic Scaling in Photoresponsive Polymer Films
Source: ACS Photonics. 2026 May 16;13(11):3081–7. doi: 10.1021/acsphotonics.5c03137 (PMC13237817; doi:10.1021/acsphotonics.5c03137)
Supplement: Supplementary file 1 [file ph5c03137_si_001.pdf]

Supporting Information for Light-driven Topological Relaxation and Dynamic Scaling in Photoresponsive Polymer Films

Michael de Oliveira[1], Sara Moujdi[1], Stefano Chiodini[1], Fabio Borbone[2], and Antonio Ambrosio[1]

[1]Center for Nano Science and Technology, Fondazione Istituto Italiano di Tecnologia, Via Rubattino 81, 20134 Milano, Italy
[2]Department of Chemical Sciences, University of Naples "Federico II", Via Cintia, 80126 Naples, Italy

S1 Experimental setup

A schematic of the experimental setup is provided in Fig. S1(a). A 488 nm writing beam (Coherent OBIS) is weakly focused using a 100 mm plano-convex lens to produce a 200 μm beam waist at the sample plane. We use a linearly polarized writing beam, with the polarization set to vertical by a half-wave plate. In this configuration, the dominant surface-relief modulation is oriented such that its wavevector is approximately parallel to the incident polarization, meaning that the periodic ridges run perpendicular to the polarization axis. This polarization selectivity follows directly from the photoisomerization probability of the elongated isomers. It is a common feature of light-driven pattern formation in these systems, although the underlying mechanisms remain an active subject of discussion in the literature.

The sample consists of an azopolymer film spin-coated onto a standard microscope coverslip, mounted flat on a XY translation stage. For each illumination condition, the sample is shifted laterally to expose an unstructured region of the film, ensuring repeatable and consistent optical conditions across different measurements. The optical alignment and detection path remain fixed throughout all experiments.

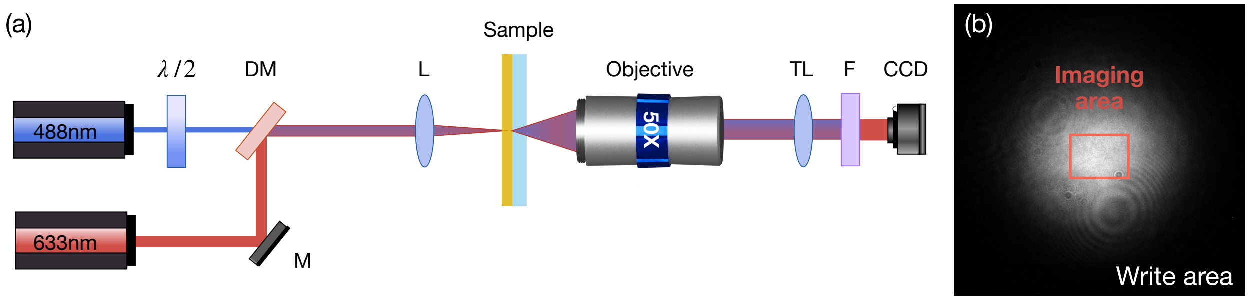

Figure S1: Single-beam illumination setup used for azopolymer self-structuring. (a) Schematic of the setup for real-time monitoring of light-induced surface patterning. A 488 nm writing beam is weakly focused onto the sample with a 100 mm lens (L), with polarization controlled via a half-wave plate ($\lambda/2$). A 633 nm He-Ne probe laser is introduced coaxially via a dichroic mirror (DM) and imaged onto a CCD camera, through a custom microscope consisting of an objective and tube lens (TL). A long-pass filter (F) blocks the writing beam. M, mirror. (b) Imaging area (75 $\times$ 100 μm^2) centered in the uniform-intensity region of the writing beam.

To monitor the surface evolution in real time, a low power 633 nm He-Ne laser (Thorlabs HNL210L) was introduced coaxially with the write beam via a dichroic mirror (Thorlabs DMLP550). Since this wavelength lies outside the azopolymer's absorption band, it does not contribute to the photoinduced patterning. The transmitted probe beam is collected through a custom microscope composed of a 50$\times$ long-working-distance objective and a 200 mm tube lens, which relays the image onto a CMOS camera (Thorlabs CS165MU). Figure S1(b) illustrates the size and position of the imaging window (75 $\times$ 100 μm^2), located at the center of the writing region where the beam intensity is uniform. A long-pass optical filter (Thorlabs FELH550) positioned before the camera blocks the residual 488 nm light, ensuring that only the probe beam is detected.

The writing beam power is varied between 5 mW to 50 mW in discrete steps. For each power setting, a new region of the sample is exposed. During each exposure, images of the evolving surface are continuously recorded and processed frame by frame in real time.

To characterize the onset and subsequent evolution of the periodic surface modulation, we represent the measured pattern in terms of a dominant carrier wavevector modulated by a slowly varying complex envelope, $\Psi(\mathbf{r}) = A(\mathbf{r})e^{i\phi(\mathbf{r})}$. The amplitude, A, quantifies the local strength of the periodic modulation, whereas its phase, ϕ, specifies the local grating phase, equivalently the local displacement of the stripe pattern relative to an ideal periodic reference.

The complex envelope $\Psi(\mathbf{r})$ is retrieved by complex demodulation of the recorded probe images. In this procedure, the 2D Fourier transform of the recorded intensity contains three main components: a central DC peak (zero-order terms) and two symmetrically displaced carrier components at spatial frequencies k_1 and $-k_1$. These side lobes correspond to the first-order diffraction from the periodic surface modulation and carry the full complex field information of the sample surface. One of the side lobes is digitally isolated and

shifted to the origin in Fourier space. An inverse Fourier transform then yields a slowly varying complex field, $\Psi(x, y)$. This procedure provides a reference-free method for reconstructing the complex envelope of periodic surface patterns, enabling quantitative, real-time mapping of order and topological defects during the self-organization process. An analogy to off-axis holography is methodological: in both cases a carrier shift separates the complex information in Fourier space, enabling its recovery by filtering. In our case, however, that carrier is generated by the self-organized grating itself rather than by an external reference.

Within this representation, topological defects appear as singular points of Ψ at which the amplitude is locally suppressed and the phase exhibits a winding of $\pm 2\pi$. These singularities therefore correspond to dislocations of the material surface-relief grating rather than optical vortices in the incident writing beam. The reconstructed phase field thus provides a direct way to locate and track defect nucleation, motion, and annihilation during pattern formation.

The first-order diffraction efficiency is calculated by integrating the intensity contained within the isolated first-order Fourier lobe. As the surface modulation develops, the first-order peak increases and saturates once the pattern approaches a steady state. Illumination was terminated at this stage, providing a consistent stopping criterion across all writing-beam powers. Importantly, the diffraction efficiency is sensitive not only to modulation depth but also to spatial phase coherence. For an ideally ordered grating, the phase of the modulation is nearly uniform over the field of view, so contributions add constructively and the diffracted power is maximized. By contrast, phase disorder, domain mismatch, and topological dislocations introduce destructive interference, reducing the net first-order signal. The first-order diffraction efficiency therefore serves as a convenient global measure of the emergence of order and provides the operational basis for defining the freeze-out time.

S2 Extracting defect positions from phase maps

To analyze the spatial distribution and properties of topological defects, we begin by retrieving the complex field corresponding to the azoplolymer surface morphology using complex demodulation, as described above and in the main text. This reconstruction yields both amplitude and phase maps, where the phase encodes key information about the local topological structure. In particular, phase singularities—points where the phase is undefined and around which it winds by integer multiples of 2π—correspond to vortices (positive topological charge) and antivortices (negative charge) in the film.

These singularities are characterized by a topological charge q, which quantifies the net phase winding around a closed path $\mathcal{C}$ encircling the singularity:

$$q = \frac{1}{2\pi} \oint_{\mathcal{C}} \nabla\phi \cdot d\mathbf{l} \tag{S1}$$

where ϕ is the local phase. The sign of q determines the chirality of the defect, while the magnitude corresponds to the number of 2π phase windings.

To compute q efficiently across a discretized phase map, we approximate the phase gradient $\nabla\phi$ as a discrete wavevector field $\mathbf{k} = \nabla\phi$. This allows us to employ a convolution-based formulation of the line integral, allowing for robust and efficient detection over large datasets. Since the curl of $\nabla\phi$ is zero in regions where ϕ is smooth, non-zero values arise only at singularities, where phase circulation is localized. The topological charge at each point can thus be computed as:

$$q(x, y) = \frac{1}{2\pi} \left[\nabla_x \otimes \mathbf{k}_y + \nabla_y \otimes \mathbf{k}_x\right]. \tag{S2}$$

Here, $\mathbf{k}_x = \frac{\partial\phi}{\partial x}$ and $\mathbf{k}_y = \frac{\partial\phi}{\partial y}$ are computed using centered finite-difference approximations, and $\otimes$ denotes 2D convolution. To avoid false singularity detection due to phase discontinuities (e.g., abrupt jumps from -π to π), we apply phase unwrapping corrections, replacing any apparent jumps exceeding π with their 2π-complement. This correction preserves continuity in the phase field and accurate gradient estimation. The operators ∇_x and ∇_y are Sobel kernels that approximate spatial derivatives in the horizontal and vertical directions, respectively:

$$\nabla_x = \begin{bmatrix} -1 & 0 & 1 \\ -2 & 0 & 2 \\ -1 & 0 & 1 \end{bmatrix}, \quad \nabla_y = \begin{bmatrix} -1 & -2 & -1 \\ 0 & 0 & 0 \\ 1 & 2 & 1 \end{bmatrix} \tag{S3}$$

These kernels operate over 3×3 neighborhoods, effectively computing the discrete analog of the line integral in Eq. (S1) by capturing the net phase circulation within each window.

The result of the convolution is a map of topological charge density, where non-zero values indicate the presence of defects. These values are rounded to the nearest integer multiple of 2π, yielding discrete topological charges $q \in \pm 1$. The spatial coordinates of each defect are extracted by locating the centroids of these localized charge distributions, and the corresponding sign assigns their chirality (vortex or antivortex).

This approach allows for automated, high-throughput identification of topological defects across the entire field of view. It is computationally efficient and robust to noise due to the integral nature of the topological charge. The resulting defect positions and charges form the basis for our statistical and spatial analyses presented in the main text.

S3 Power-Dependent Surface Structuring of Azopolymers

Surface patterning in azopolymers is typically achieved through interference-based exposure, where periodic fringe patterns dictate the resulting surface modulation. In these conventional schemes, the structural periodicity is fixed by the interference geometry, limiting dynamic tunability and requiring coherent beam setups. In contrast, our approach employs a single-beam, without any engineered spatial modulation, to induce self-organized surface relief structures in the high-intensity regime, circumventing the need for structured light or interferometry.

We characterized the steady-state topography of the azopolymer surface after exposure to varying illumination powers using atomic force microscopy (AFM). The AFM topographies, shown in Fig. S2(a-c), for 15 mW, 30 mW, and 45 mW reveal clear periodic structuring with a rectified, sinusoid-like profile. Although the AFM scan area (5 x 10 μm^2) is much smaller than the optical field-of-view used for real-time imaging, the AFM measurements corroborate the observed trend in the main text: lower powers result in more disordered and defect-laden structures, while higher powers yield increasingly uniform, periodic patterns.

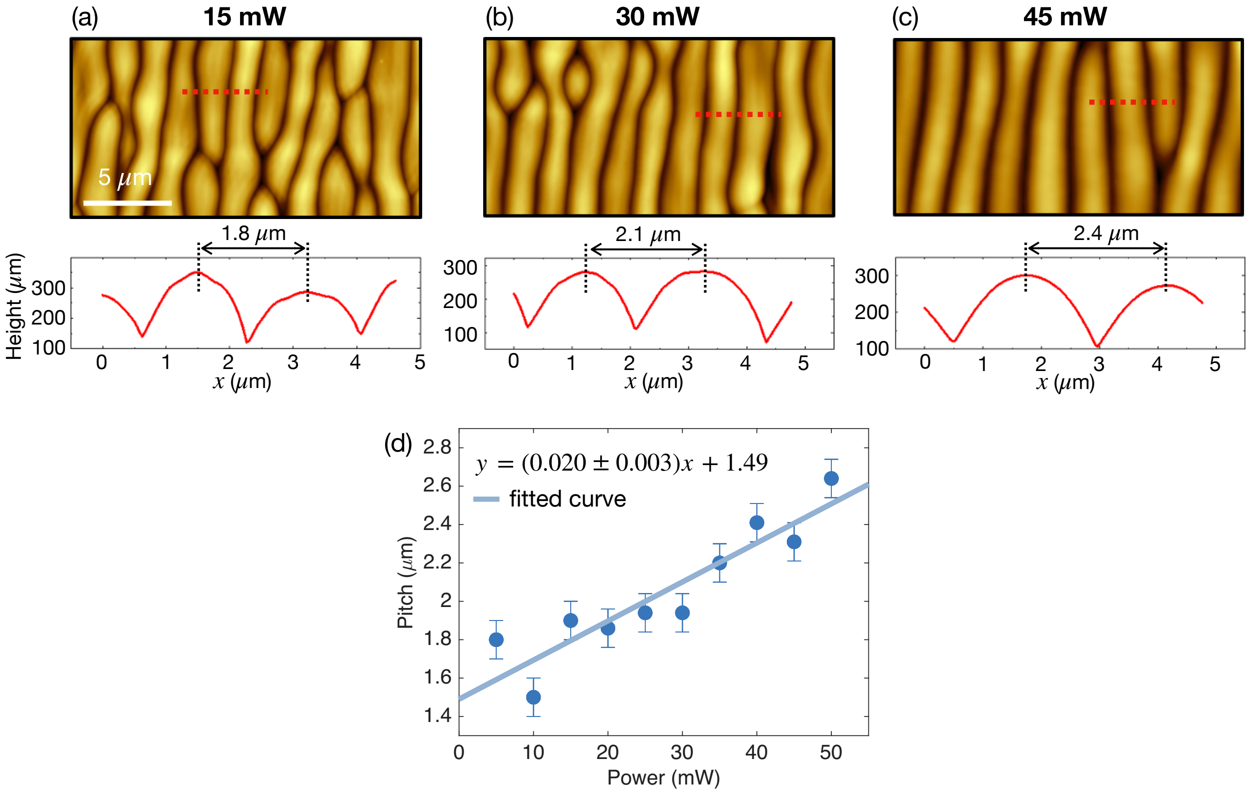

Figure S2: AFM characterization of steady-state surface patterns. (a–c) AFM topography maps of the azopolymer surface after exposure to 15 mW, 30 mW, and 45 mW writing beams, respectively. All images show clear periodic structuring, with improved uniformity at higher powers. Line profiles (below) correspond to the red dashed traces. Scan area: 5×10 μm^2. (d) Extracted average periodicity versus illumination power, revealing tunable surface pitch controlled by optical intensity.

From the AFM profiles, we extract the spatial period of the surface structures and plot the average periodicity as a function of illumination power in Fig. S2(d). A linear relationship is observed, with a fitted slope of 0.020 ± 0.003 μm/mW, corresponding to a near-doubling of the period—from 1.50 μm at 10 mW to 2.64 μm at 50mW. This result demonstrates active tunability of the pattern periodicity via optical power of a uniform beam, without the need for spatial light modulation or interferometric setups.

This result is significant in several respects. First, it confirms that periodic azopolymer structuring can be achieved using homogeneous light fields, with the feature size governed not by the spatial distribution of

the intensity, but by the magnitude of the optical power. This points to a self-organization mechanism that is governed by light-driven mass transport coupled with nonlinear photo- and thermo-mechanical effects. Second, the observed power-dependent periodicity offers a simple and scalable route to tunable surface patterning, without the need for beam shaping, interferometry, or lithographic masks. This approach enables rapid prototyping and reconfiguration of surface features over large areas, making it particularly attractive for applications in adaptive optics, beam shaping, and optical surface engineering, where dynamic control of structural parameters is advantageous. Finally, the ability to modulate both the periodicity and the degree of structural ordering via a single optical parameter (illumination power) adds a useful degree of freedom for the design of reconfigurable photonic interfaces, toward self-organized defect minimization, which may be harnessed to improve optical quality or to engineer defect distributions for functional metasurfaces.

S4 Write–erase–rewrite test of pattern reversibility

To test whether the observed defect dynamics are reversible under optical driving, we performed a write–erase–rewrite measurement on the same region of the azopolymer film. The film was first illuminated as before at 50 mW for 160 s, then erased by increasing the illumination power to 70 mW for 30 s, and finally rewritten by returning to 50 mW for a further 160 s.

During the initial writing stage, the first-order diffraction signal increased while the defect number decreased. During the erasure step, the diffraction signal dropped to near the noise floor and the defects disappeared, consistent with recovery of an approximately uniform surface state. When the power was returned to 50 mW, the diffraction signal increased again and defects reappeared as the quasi-periodic modulation re-formed. In both writing stages, the diffraction efficiency and defect density followed the same two-stage evolution described in the main text.

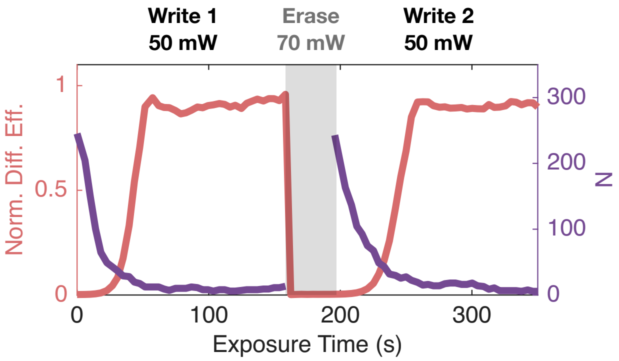

Figure S3: Write–erase–rewrite measurement demonstrating reversible pattern formation. A single region of the azopolymer film was written at 50 mW for 160 s, erased at 70 mW for 30 s, and rewritten at 50 mW for a further 160 s. Time evolution of the defect number N (purple) and diffraction efficiency (red) during the full sequence.

These observations show that the pattern can be optically erased and re-established in the same region of the film, supporting the interpretation that the measured scaling and coarsening arise from a reversible driven nonequilibrium process rather than from a one-time irreversible surface imprint of a randomly nucleated surface-relief pattern. We note, however, that repeated cycling may also be affected by cumulative fatigue, residual stress, or partial mass redistribution associated with imperfect recovery of the initial flat film state.

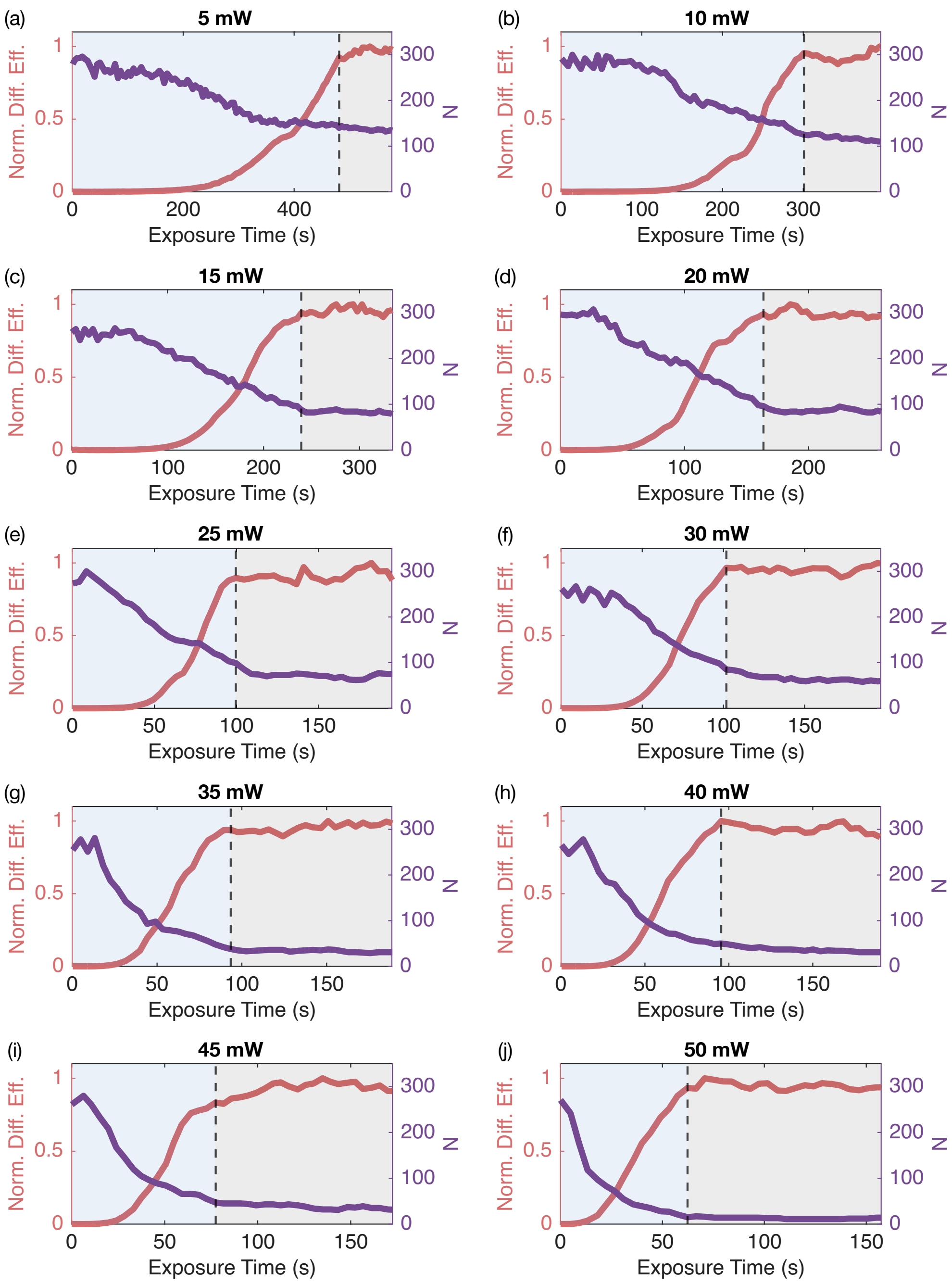

Figure S4: Supplementary analysis of power-dependent defect dynamics. (a–j) Time evolution of defect number N (purple) and diffraction efficiency (red) for illumination powers from 5 to 50 mW. The freeze-out time t_f (black dashed line) is defined by the inflection point of the diffraction efficiency curve. This point also corresponds to a noticeable change in the slope of the defect curve. A secondary regime (gray shaded) follows, characterized by decreasing defect number and a linear increase in diffraction efficiency. The non-monotonic dynamics suggest ongoing spontaneous creation and annihilation as the system relaxes toward lower energy configurations.

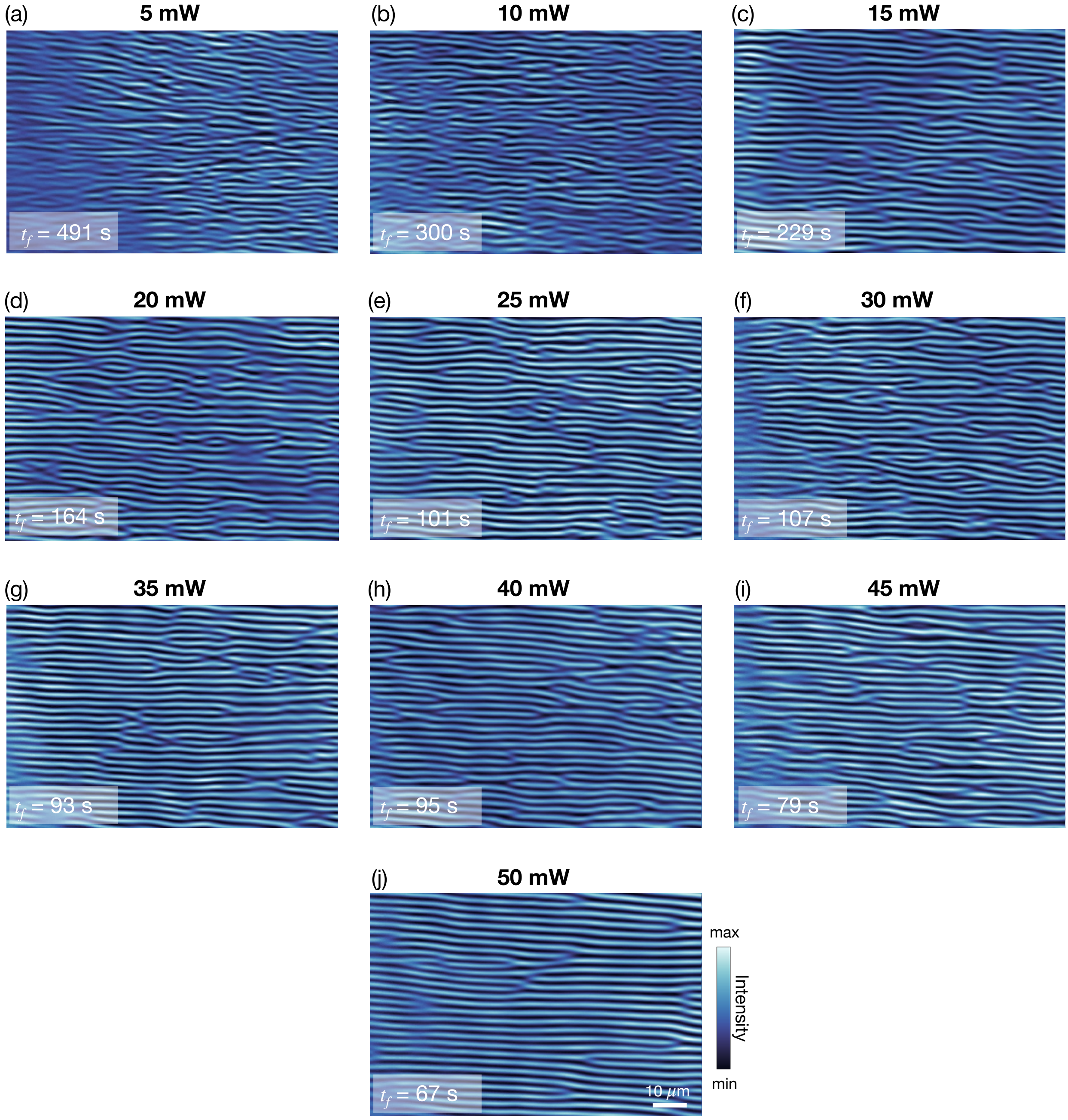

Figure S5: Surface morphology at the freeze-out time t_f for varying illumination powers. Optical imaged of the azopolymer surfaces captured at t_f for illumination powers ranging from 5 to 50 mW. At low powers, the morphology is highly irregular and densely populated with topological defects. As power increases, the surface becomes progressively more uniform, with fewer defects and greater spatial coherence, indicating more ordered domain formation.

Video S1 Real-time evolution of the surface morphology of the azopolymer under 15 mW illumination, recorded at 1 frame per second. Periodic ripples emerge across the surface, interspersed with characteristic pitchfork-like topological defects. The field of view is 75×100 μm^2 (MP4, 14.6MB).
